# Reducing Coercive Field and Improving Endurance in Ferroelectric Epitaxial Hf_0.5_Zr_0.5_O_2_ Thin Films via Novel Interface Layer Approach

**DOI:** 10.1002/advs.202517314

**Published:** 2025-11-14

**Authors:** Ji Soo Kim, Benedetta Gaggio, Babak Bakhit, Veniero Lenzi, Luis Marques, Simon M. Fairclough, Nives Strkalj, Duk‐Hyun Choe, José P. B. Silva, J. L. MacManus‐Driscoll

**Affiliations:** ^1^ Department of Materials Science & Metallurgy University of Cambridge 27 Charles Babbage Road Cambridge CB3 0FS United Kingdom; ^2^ Physics Center of Minho and Porto Universities (CF‐UM‐UP) University of Minho Campus de Gualtar Braga 4710‐057 Portugal; ^3^ Laboratory of Physics for Materials and Emergent Technologies, LapMET University of Minho Braga 4710‐057 Portugal; ^4^ Samsung Advanced Institute of Technology Samsung Electronics Suwon‐si 16678 South Korea; ^5^ Present address: Center for Advanced Laser Techniques Institute of Physics Zagreb 10000 Croatia

**Keywords:** ferroelectrics, hafnia, epitaxial films, non‐volatile memory devices

## Abstract

Ferroelectric doped hafnium oxide (HfO_2_) has emerged as CMOS‐compatible and scalable ferroelectric for next‐generation memory/in‐memory computing devices. However, its high coercive field (E_c_) and limited endurance remain key obstacles. Here, a ≈25% reduction in E_c_ from 3.3 to 2.5 MV/cm and an order of magnitude increase in endurance by implementing an ultrathin (≈2 nm) 5 at.% Sm‐doped HZO (HZSO) ionic conducting underlayer for HZO are shown. X‐ray photoelectron spectroscopy (XPS) results reveal the absence of redox effects during primary ferroelectric switching in HZSO, unlike in HZO. NEB calculations show that V_O_‐rich HZSO lowers the switching barrier compared to that of HZO, which agrees with experimental results. Notably, these improvements are achieved in HZSO|HZO without compromising P_r_ compared to HZO. This approach presents a new powerful route to engineering ferroelectric properties in doped HfO_2_, applicable to both epitaxial and polycrystalline films for future memory devices.

## Graphical abstract

An ultrathin samarium doped hafnium zirconium oxide (Sm:HZO) underlayer reduces coercive field by ∼25% and enhances endurance tenfold in epitaxial ferroelectric HZO overlayer. The Sm:HZO interface allows redox mitigated oxygen exchange and lowers switching barriers, achieving stable polarization with enhanced endurance performance. This interfacial engineering strategy offers a scalable route for low power ferroelectric memory applications.

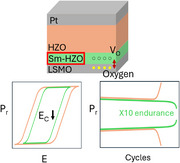

## 1 Introduction

Ferroelectricity has always been an attractive phenomenon in non‐volatile memory devices.^[^ ^1^ ^]^ The discovery of ferroelectricity in complementary metal‐oxide‐semiconductor (CMOS) compatible HfO_2_‐based oxides^[^ ^2^, ^3^, ^4^ ^]^ has excited the ferroelectric community, motivating further extensive studies on the fundamentals and novel applications of ferroelectric HfO_2_ thin films, such as devices for energy‐efficient memory devices, neuromorphic computing devices, and energy storage devices.^[^ ^1^, ^2^, ^5^, ^6^, ^7^, ^8^, ^9^ ^]^

Although ferroelectric HfO_2_ is attractive from a CMOS compatibility viewpoint, it nevertheless has challenges, particularly in the polycrystalline form.^[^ ^5^ ^]^ On the other hand, by stabilizing (near‐)single phase films through epitaxial growth of polar doped‐HfO_2_, one learns more on the fundamental behaviour for improving ferroelectric properties in doped‐HfO_2_ systems. Epitaxial HfO_2_ thin films, typically made by pulsed laser deposition (PLD), can exhibit one of the polar phases among orthorhombic (*o‐*) (P*ca*2_1_),^[^ ^10^, ^11^, ^12^, ^13^ ^]^ rhombohedrally distorted orthorhombic (*r‐*d *o*‐) (P*ca*2_1_),^[^ ^14^, ^15^, ^16^ ^]^ and rhombohedral (*r‐*) (R3 or R3*m*).^[^ ^17^, ^18^ ^]^ One of the parameters implemented to improve ferroelectric properties is doping,^[^ ^19^ ^]^ especially acceptor doping with dopants such as Sr,^[^ ^20^ ^]^ Ca,^[^ ^21^ ^]^ Y,^[^ ^14^ ^]^ La,^[^ ^22^, ^23^ ^]^ and Al.^[^ ^24^, ^25^ ^]^ It is understood that the induced oxygen vacancy (V_O_) from acceptor doping lowers the energy barrier to stabilize the ferroelectric phase,^[^ ^26^, ^27^, ^28^ ^]^ indicating the important role of V_O_ in the ferroelectric properties of doped HfO_2_. While acceptor dopants are beneficial for V_O_ formation, they often enhance trap‐assisted electron transport^[^ ^29^ ^]^ or undesirable band alignment at the interface, which can degrade their ferroelectric performance.^[^ ^30^ ^]^

Generally, epitaxial ferroelectric doped HfO_2_ exhibits a higher coercive field (E_c_) than polycrystalline films, and this can cause relatively easier dielectric breakdown.^[^ ^31^, ^32^ ^]^ This is not only observed in PLD‐grown systems, but also in industry‐compatible atomic‐layer‐deposition‐(ALD)‐grown HfO_2_‐based films where the E_c_ could exceed 3 MV/cm.^[^ ^33^ ^]^ Therefore, there are several ongoing attempts to reduce E_c_ further in polycrystalline ferroelectric HfO_2_‐based thin films such as electrode engineering,^[^ ^34^ ^]^ superlattices,^[^ ^35^ ^]^ doping,^[^ ^36^ ^]^ etc. Furthermore, high E_c_ and inevitable formation of V_O_ (for polar phase stabilization) in doped‐HfO_2_ systems have a strong correlation with the fatigue performance of HfO_2_. In epitaxial films, irrespective of the phase, HfO_2_‐based films generally show fatigue behaviour (here defined as less than 80% of initial P_r_) at <10^7^ cycles.^[^ ^14^, ^16^, ^18^, ^23^, ^37^, ^38^, ^39^, ^40^, ^41^, ^42^, ^43^ ^]^ Usually, this behavior is associated with the uncontrolled reversible extraction and insertion of oxygen anions into the HfO_2_‐based film by the La_0.7_Sr_0.3_MnO_3_ electrode.^[^ ^44^ ^]^ In Hf_0.5_Zr_0.5_O_2_ (HZO), the most explored HfO_2_‐based material due to its high ferroelectric polarization, formation of V_O_ is associated with cation redox effects. This renders reversible switching of polarization more complex since O defect dynamics can lead to irreversible phase change to nonpolar phases.^[^ ^45^, ^46^ ^]^ These effects can impede the oxygen insertion/extraction, and can lead to fatigue. Therefore, in order to improve the endurance performance of ferroelectric HfO_2_‐based films, it is important to develop various strategies such as controlling the concentration of V_O_ for phase stability during prolonged cycling and allowing V_O_ to move in facile and cyclable ways between the ferroelectric and the O source electrode,^[^ ^47^, ^48^, ^49^ ^]^ as well as exploiting electrostatic effects using dielectric capping layers.^[^ ^50^ ^]^

Here, we hypothesize that if HfO_2_‐based thin films have an underlayer of aliovalent‐doped HfO_2_ with a controlled concentration of structural V_O_ with lower E_c_ than HZO, E_c_ in HZO could be reduced by a proximity effect. The ferroelectric polarization in HZO layer would switch at a lower electric field when placed in close proximity to a ferroelectric doped‐HfO_2_ layer with lower E_c_, similar to the proximity effect observed in nitride‐ based ferroelectrics.^[^ ^51^ ^]^ In addition, O can more readily exchange between acceptor‐doped HfO_2_ and LSMO. Together, these factors could make the system fatigue more slowly. By implementing the above hypothesis, i.e., by using a 5 at.% Sm‐doped HZO (HZSO) ionic conductor^[^ ^52^ ^]^ underlayer for HZO thin films on LSMO on SrTiO_3_ (STO), we demonstrate a reduced E_c_ by ≈25% and improved endurance performance by an order of magnitude. A 5% dopant concentration of Sm is deemed optimal based on previously reported epitaxial ferroelectric acceptor‐doped HfO_2_ thin films such as La‐HfO_2_ (2–5%)^[^ ^53^, ^54^ ^]^ and Y‐HfO_2_ (≈7%).^[^ ^14^, ^55^ ^]^ Also, recently reported epitaxial ferroelectric Smdoped HfO_2_ showed the highest polarizationsat 3.5‐7.5% of Sm doping concentration. Therefore, with 5% of Sm doping in HZO, both strong ferroelectric polarization and high structural V_O_ concentration can be achieved, enabling plentiful of sites for O exchange at the LSMO|HZSO interface.

## 2 Results and Discussion

HZO and HZSO samples with a thickness of ≈10 nm were deposited on La_0.7_Sr_0.3_MnO_3_‐(LSMO)‐buffered TiO_2_ terminated SrTiO_3_ (STO) (001). A ≈10 nm thick HZSO|HZO bilayer was deposited on the identical substrate consisting of an HZO film and an HZSO seed layer with ≈8 nm and ≈2 nm of thickness, respectively. The HZSO underlayer is a suitable homoepitaxial template for the growth of HZO, benefiting from a similar lattice parameter, similar composition and the same structure as HZO. Therefore, we could minimize formation of defects which are detrimental to endurance.

**Figure 1:**
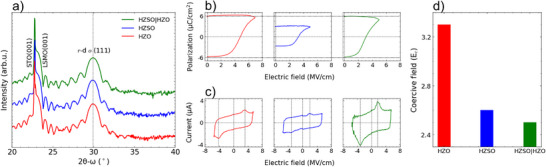
a) 2θ‐ω x‐ray diffraction scans of HZO (red), HZSO (blue), and HZSO|HZO (green) thin films on STO|LSMO substrate. Stabilization of near‐single *r*‐d *o*(111) phase was observed from all the samples. b) Polarization‐electric field (P‐E) plots from positive‐up (PU) measurement using tungsten (W) top electrode. HZSO required 7 MV cm^−1^ for complete saturation of polarization. c) Current–electric field (*I*–*E*) plots from dynamic leakage current compensation (DLCC) measurement. Sm doping and Sm‐the doped HZO seed layer reduce coercive field (E_c_). P_r_ is reduced in HZSO, but not in HZSO|HZO. d) Plot showing change in E_c_ for HZSO (blue) and HZSO|HZO (green) compared to HZO (red).

In order to characterize the ferroelectric phase in the thin films, x‐ray diffraction (XRD) scans along the 2θ‐ω axes are performed, see **Figure** **1**a. The diffraction peak at ≈29.9° (<30°) corresponds to the (111)‐oriented HZO film with rhombohedrally‐distorted orthorhombic (*r*‐d *o*) phase.^[^ ^14^, ^16^ ^]^ 2θ scans performed at χ ≈70° and ϕ ≈45°, which allows us to access the in‐plane lattice parameter of *r*‐d *o*(111), see Figure S1a (Supporting Information). Rhombohedral distortion in *o*‐phase was confirmed from the in‐plane 2θ peak position differing from the out‐of‐plane 2θ peak position, indicating d_111_ ≠ d_11 − 1_.^[^ ^14^ ^]^

The films were electrically tested with tungsten (W) top electrodes. They did not show any wake‐up behavior, typical of epitaxial films.^[^ ^17^, ^56^ ^]^ In Figure 1b, fully saturated polarization‐electric field (P‐E) measurements are shown. P‐E was performed using positive‐up (PU) measurement, allowing us to measure their polarization value solely from the ferroelectric displacement contribution, disregarding leakage current.^[^ ^2^ ^]^ HZO required a higher voltage (7 V) due to its high E_c_ to achieve a full saturation of polarization. It is observed that the polarization value is reduced by half from ≈6 $\mu {C/\mathrm{cm}}^{2}$ to ≈3 $\mu {C/\mathrm{cm}}^{2}$ in HZSO compared to HZO, but the HZSO|HZO film of interest retains the same P_r_ as HZO. Figure 1c shows current–electric field (*I*–*E*) plots from dynamic leakage current compensation (DLCC) measurements with clear ferroelectric switching peaks, which are used to obtain the E_c_ values.^[^ ^57^ ^]^ Fundamentally, PU measurement and DLCC measurements are different as PU excludes leakage and dielectric contributions while DLCC only excludes frequency‐independent (leakage) contributions. However, frequency‐dependent leakage current associated with ionic conduction cannot be eliminated and may contribute to the measured polarization values. For this reason, the P–E loops and the I–E curves shown in Figure 1c,d were measured at different maximum applied electric fields. Nevertheless, the fully saturated P_r_ extracted from both measurement types are comparable. Figure 1d shows the E_c_ values of HZO, HZSO, and HZSO|HZO in bar plots. E_c_ values were taken from the non‐linear ferroelectric switching peaks from DLCC measurements. HZSO exhibits E_c_ ≈2.6 MV/cm, ≈20 % reduction of E_c_ from the HZO value of 3.3 MV/cm. A similar, slightly larger, reduction to 2.5 MV/cm in E_c_ was observed in HZSO|HZO, indicating the reduction of E_c_ in HZO could be triggered with the help of the low E_c_ in the HZSO seed layer. Such behaviour resembles the proximity ferroelectricity effect observed in nitride‐based ferroelectric films.^[^ ^51^ ^]^

The laser fluence used to grow the HZSO layer in the HZSO|HZO bilayer is 0.5 J/cm^2^, which is lower than the 1.3 J cm^−^2 used to grow the HZO monolayer film (see Experimental Section). Initially, the lower fluence was chosen to ensure the highest quality layer to act as a seed for the HZO above. It is important to elucidate the influence of laser fluence in the stabilization of the *r*‐d *o*(111) phase and physical properties (E_c_ and P_r_); see Figure S1b– d (Supporting Information). Here, we find a stabilization of *r*‐d *o*(111) phase regardless of the laser fluence. Also, HZSO grown at a lower laser fluence (0.5 J cm^−2^) exhibits ≈2.6 MV/cm of E_c_ and ≈3 μC cm^−2^ of P_r_, which is identical to the HZSO grown at higher laser fluence as shown in Figure 1b–d. Therefore, it is concluded that the physical properties of HZSO do not change with laser fluence; thus, P_r_ and E_c_ are intrinsic properties induced by Sm doping in HZO.

**Figure 2:**
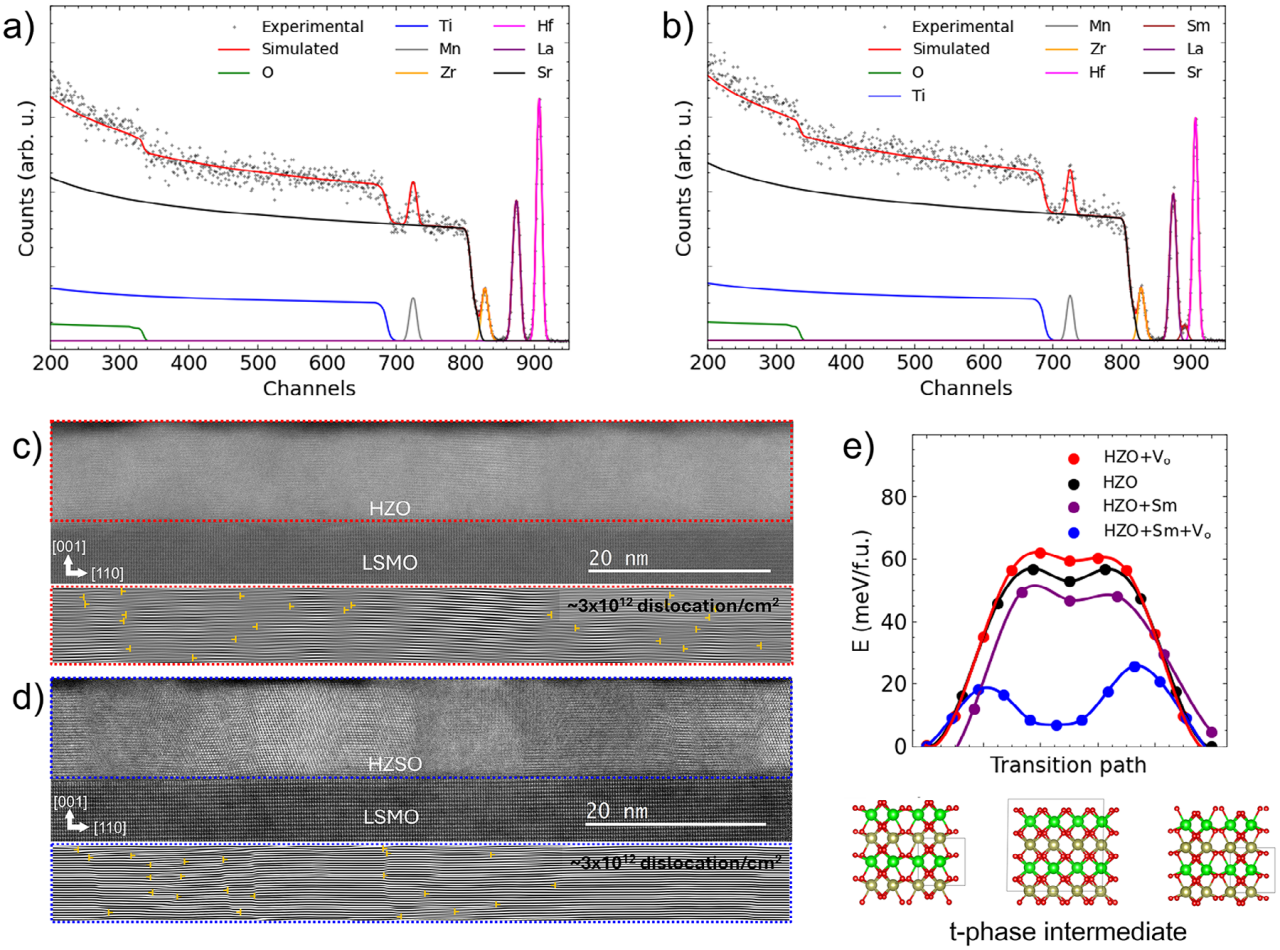
Rutherford Backscattering Spectroscopy (RBS) on a) STO|LSMO|HZO and b) STO|LSMO|HZSO. The stoichiometries of HZO and HZSO are determined to be Hf_0.53_Zr_0.47_O_1.72_ and Hf_0.5_Zr_0.45_Sm_0.05_O_1.61_. Cross‐sectional high angle annular dark field (HAADF) images along zone axis [110] STO, ϕ = 45° for c) HZO and d) HZSO. Dotted colored boxes in HAADF images are the regions where Inverse Fast Fourier Transforms (IFFT) were performed to identify dislocation density of each film. Yellow cross markers are dislocations. Both HZO and HZSO show ≈3 × 10^12^ dislocation/cm^2^ of dislocation density. e) Nudge Elastic Band (NEB) calculations for minimum ferroelectric switching energy for various conditions: HZO (black), HZO with V_O_ (red), and HZO with Sm (purple) at 6.25 f.u.%, HZO with Sm at 6.25 f.u.%, and V_O_ (blue) at 3.125 f.u.%.

Rutherford Backscattering Spectroscopy (RBS) was performed on STO|LSMO|HZO and STO|LSMO|HZSO to determine the chemical composition of the layers, see **Figure** **2**a,b. The oxygen‐deficient compositions of HZO and HZSO are determined to be Hf_0.53_Zr_0.47_O_1.72_ and Hf_0.5_Zr_0.45_Sm_0.05_O_1.61_, respectively. First, we note that the HZO layer is not stoichiometric in O, likely because the LSMO layer depleted the O,^[^ ^47^, ^58^ ^]^ and the strain effects from domain matching epitaxy of two dissimilar interfaces.^[^ ^59^ ^]^ For the HZSO layer, each Sm^3 +^ ion on a Hf^4 +^/Zr^4 +^ site is expected to induce $\frac{1}{2}V$_O_ for charge compensation. Thus, 0.05 Sminduces 0.025 V_O_^2 +^ and thus the O content in HZO is expected to decrease from 1.72 to 1.67, in general agreement with our observation of 1.61.

To investigate whether the reduction in E_c_ in HZSO is caused by a reduction in the density of defect pinning sites, a dislocation density analysis based on scanning tunneling electron microscopy (STEM) was performed along the zone axis [110] STO. Our previous study on HZO suggested that E_c_ has a strong correlation with the density of domain wall‐pinning dislocations in HZO films, which increases for higher laser fluences,^[^ ^56^ ^]^ such effects being well‐established in ferroelectric films.^[^ ^60^, ^61^, ^62^, ^63^ ^]^ In Figure 2c,d, cross‐sectional high‐angle annular dark‐field (HAADF) image and inverse Fast Fourier Transform (IFFT) of HZO and HZSO are shown. IFFT was performed on the red and blue dotted boxes from the cross‐sectional HAADF images. From IFFT, dislocations were identified, and the dislocation density of ≈3 × 10^12^ dislocation/cm^2^ was obtained for both HZO and HZSO. From an equivalent dislocation density in HZO and HZSO, we conclude that the reduced E_c_ for HZSO is not linked to a reduction in domain wall pinning by dislocations.

As reduced E_c_ in HZSO is not related to a pinning effect per dislocation density analysis, Nudge Elastic Band (NEB) calculations were performed to further investigate the source of reduced E_c_ with Sm doping. NEB calculations were performed for *r*‐d *o*‐ to tetragonal (*t*‐) and *t*‐ to *r*‐d *o*‐ polarization transition paths for various conditions, see Figure 2e. The conditions include HZO (HZO), HZO with V_O_ at 3.125 f.u.% (HZO+V_O_), HZO with Sm at 6.25 f.u.% (HZO+Sm) and HZO with Sm at 6.25 f.u.% and V_O_ 3.125 f.u.% (HZO+Sm+V_O_) in order to replicate the composition of the layers. It shows that the introduction of Sm and V_O_ in HZO can significantly reduce the energy barrier for polarization switching. It is noted that i) aliovalent ion doping of the Hf^4 +^/Zr^4 +^ site by Sm^3 +^ will induce V_O_,^[^ ^52^ ^]^ and ii) the V_O_ formation is generally favored near the dopant.^[^ ^64^ ^]^ In Figure S2a, NEB calculations are shown for HZO+Sm+V_O_ with different locations of V_O_. The energy barriers are approximately the same regardless of the location of V_O_. Thus, Figure 2e shows just an energy barrier profile for one of the V_O_ locations in HZO+Sm+V_O_. Figure S2b (Supporting Information) shows a plot of the intermediate energy barriers for each condition. It is clear that HZO+Sm+V_O_ shows less than half of the average energy barrier (<20 meV) compared to HZO or HZO+V_O_ or HZO+Sm. It is noted that in NEB calculations, relatively lower V_O_ concentration (3.125%) was employed due to constraints with computational supercell size. It nonetheless reflects relative energy barrier as extra V_O_ generated from Sm doping is ≈4.5%. Overall, the reduced energy barrier in HZO with Sm and V_O_ calculated by NEB is in agreement with the measured reduced E_c_ in HZSO.

**Figure 3:**
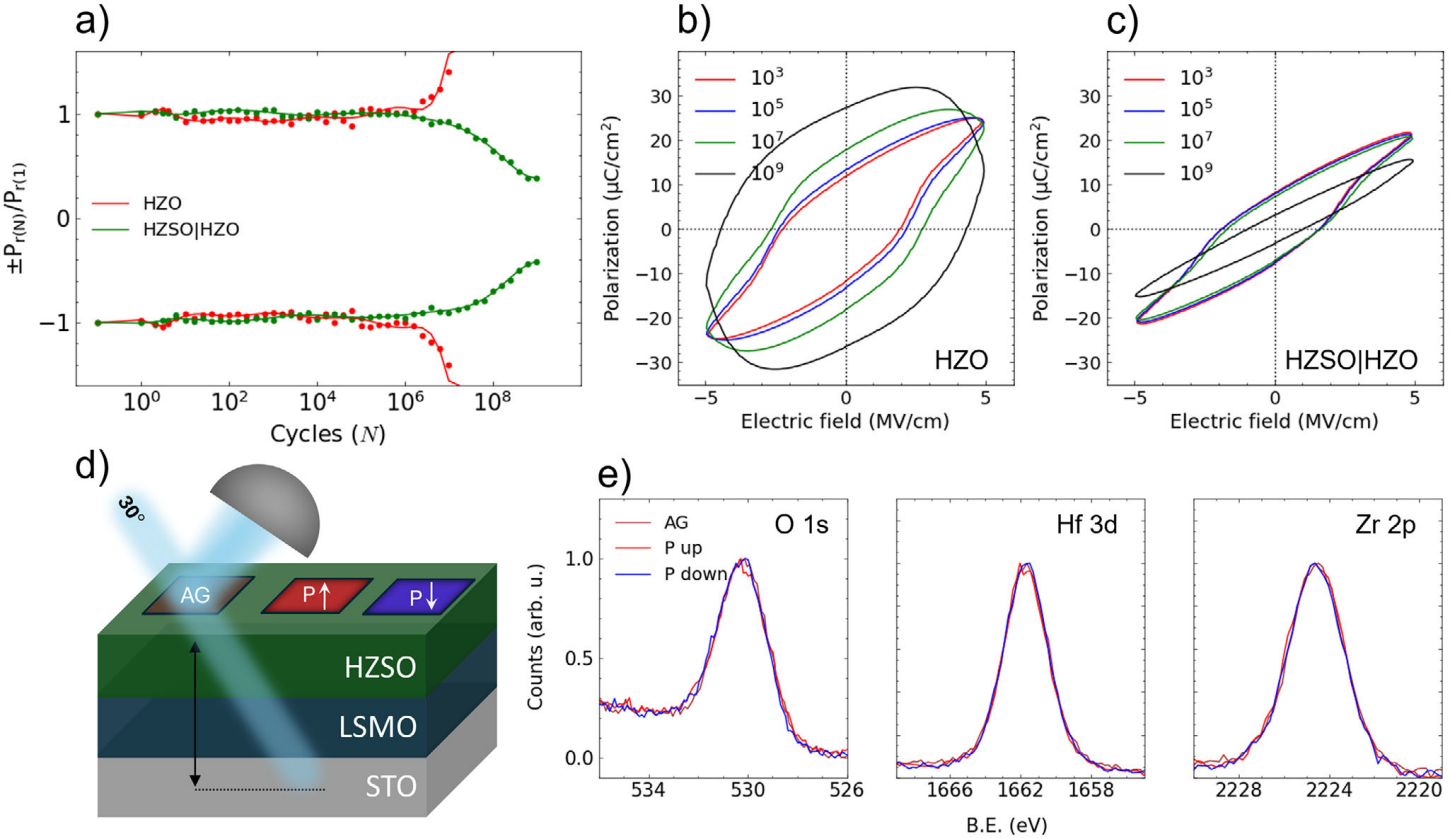
a) Endurance measurements on HZO (red) and HZSO|HZO (green). HZO demonstrates redox‐assisted electron transport fatigue. HZSO|HZO demonstrates ferroelectricity degradation with cycling. Dynamic hysteresis measurement (DHM) in the final fatigue cycle showing P–E loops of b) HZO and c) HZSO|HZO. d) Schematic of Hard X‐ray Photoelectron Spectroscopy (HAXPES) set up on 10‐nm‐thick HZSO thin film. Incident X‐ray was shone on 250×250 μm ^2^ PF‐ polarized regions with 30° of incidence angle. e) O‐1s, Hf‐3d, and Zr‐2p spectra for P up, P down, and as grown (AG) states.

To further evaluate the role of the HZSO seed layer, endurance measurements were performed with a top Pt electrode (50 μm of diameter) for HZO and HZSO|HZO. Rectangular voltage pulses were applied at 500 kHz and 1 kHz dynamic hysteresis measurement (DHM) was conducted five times every decade of cycles. In **Figure** **3**a, the fatigue behaviors of HZO and HZSO|HZO thin films are shown. It is noted that fatigue is defined as the number of cycles in which P_r_ is degraded to 80% of its initial P_r_. We recall that in order to achieve robust reversible ferroelectric polarization switching in epitaxial HZO films, it is essential to control the reversible extraction and insertion of oxygen anions into the HZO film by the LSMO.^[^ ^44^ ^]^ Endurance values of 5× 10^6^ cycles and 5× 10^7^ cycles were obtained from HZO and HZSO|HZO, respectively. For HZO, the polarization appears to increases with cycling after 5× 10^6^ cycles (Figure 3b), as a result of sharp increase in leakage current, consistent with cation redox effects.^[^ ^46^ ^]^ On the other hand, for HZSO|HZO, a gradual decay of polarization is observed (Figure 3c), indicative of gradual accumulation of V_O_ and slow conversion from polar to nonpolar phases, likely near the HZSO|LSMO interface. Leakage current measurement (Figure S3, Supporting Information) on HZSO|HZO for pre‐ and post‐fatigue measurement demonstrate clear suppression of leakage current, allowing it to fully exploit switchable polarization, unlike HZO where redox‐assisted electron transport increases the leakage current.

We then inspect the fatigue behavior of an HZSO single layer thin film of ≈10 nm thickness to deduce whether the improved fatigue in HZSO|HZO can be ascribed to HZSO. Figure S4 (Supporting Information) shows the equivalent fatigue behavior for the HZSO single layer films grown under different laser fluence of 0.5 J cm^−2^ and 1.3 J cm^−2^ to that in the HZSO|HZO bilayer film (Figure 3a). This indicates that the improved endurance is intrinsic to the Sm doping effects in HZSO.

To determine the influence of Sm‐doping in HZO on electrochemical changes under ferroelectric switching, Hard X‐ray Photoelectron Spectroscopy (HAXPES) was performed on an HZSO film of ≈10 nm thickness. In Figure 3d, a schematic of the HAXPES setup is shown. A high energy of 9.25 keV from laboratory‐based XPS with a Ga‐kα allows probing the entire HZSO thin film. The measurements were performed on three regions: P up (–8 V), P down (+8 V), and as‐grown (AG). Except for the AG region (unpoled and pristine), 250×250 μm ^2^ square regions are polarized using piezo‐response force microscopy (PFM) for the P up and P down states. Prior to HAXPES measurement, the retention of polarization in HZSO was tested to exclude the possibility of relaxation while the sample was transported to the beamline. PFM polarized regions retained their polarized states (±8 V) for more than 16 hours; see Fig. S5a‐c.

In Figure 3e, the core‐level spectra of O‐1s, Hf‐3d, and Zr‐2p for P up (red), P down (blue), and AG (black) of HZSO are shown. O‐1s, Hf‐3d, and Zr‐2p remain constant with polarization switching without any peak shift, peak broadening or emergence of secondary peaks. There is a strong correlation between redox and the emergence of non‐lattice oxygen (NL‐O) peak in O‐1s^65^ ^]^ NL‐O is a secondary peak that could arise at BE ≈532–534 eV from multiple factors such as adsorbed hydroxyl/hydroxide or interstitial oxygen/hydrogen.^[^ ^65^ ^]^ Absence of a change in NL‐O and Hf‐3d with polarization switching suggests that HZSO does not experience redox, unlike HZO where cation redox behaviour was observed.^[^ ^65^ ^]^ Thus, intrinsic polarity of HZSO without redox is consistent with it being an ionic conducting layer. Similarly, lack of redox was observed in Y‐doped HfO_2_ thin film, another aliovalent ionic conductor, where a minute rise of NL‐O was attributed to metal electrode oxide interlayer formation rather than redox of the HfO_2_‐based layer.^[^ ^32^ ^]^

The peak fittings of the O‐1s core‐level spectra for P up, P down and AG from HAXPES measurement on HZSO are shown in Figure S6a (Supporting Information). The O‐1s peak contributions and the FWHM of Hf‐3d and Zr‐2p are shown in Table S1 (Supporting Information). Minute changes were found from fitting, but those changes are within the resolution limit. It is important to note that NL‐O cannot be directly associated with V_O_ as V_O_ implies an absence of oxygen and absent species cannot cause photoemission^[^ ^66^ ^]^

In‐situ XPS with an Al Kα x‐ray source (≈1.49 keV) was also performed on HZSO and HZSO|HZO films. Immediately after growth, the films were transferred through vacuum from the PLD chamber to the XPS chamber. Owing to the low energy of the x‐rays compared to HAXPES, the probing depth is smaller (≈3–5 nm cf. >20 nm in HAXPES). It enables analysis of the uppermost HZO region of the film, and thus evaluation of possible Sm diffusion into HZO from the HZSO layer. In Figure S6b (Supporting Information), Sm‐3d spectra for HZSO (black) and HZSO|HZSO (red) are shown. While a clear doublet is shown from HZSO that confirms Sm in the film, no Sm peak was detected in the HZSO|HZO film, indicating that Sm does not diffuse to the HZO layer. This confirms that the reduced E_c_ is due to the proximity effect of the HZSO seed layer rather than reduction in energy barrier derived from the diffusion of Sm into the HZO layer. The absence of a distinguishing peak from C‐1s core‐level from in‐situ XPS is a result of an in‐situ transfer of the samples without adsorption of organic species on the surface; see Figure S6c (Supporting Information).

**Figure 4:**
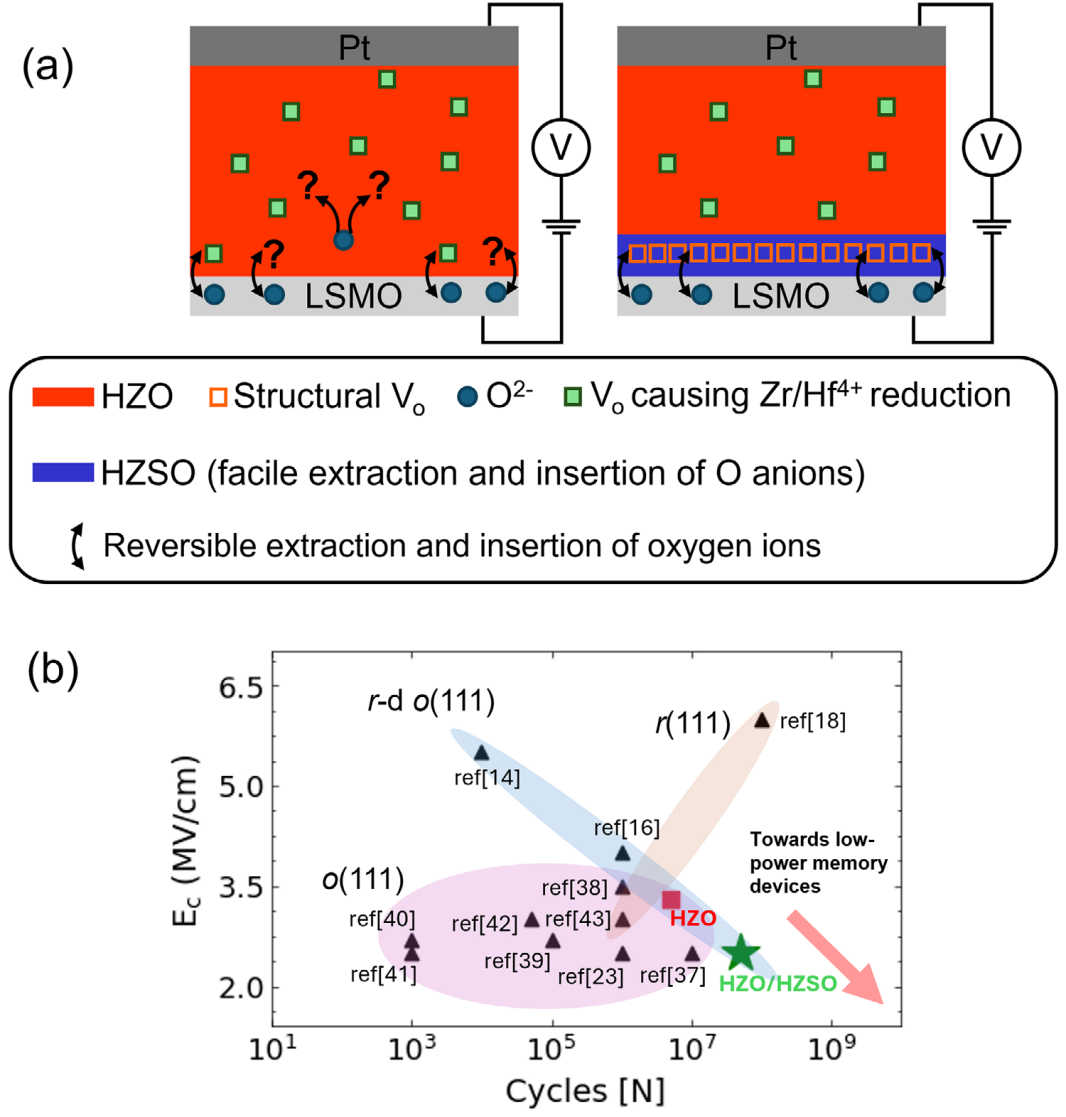
a) Schematics of insertion and extraction of oxygen anions in HZO (left) and HZSO seed layer in HZSO|HZO films (right) under cycling. The increased number of controlled structural $V_{O}$ sites in HZSO promotes the oxygen anion movement at the interface without redox effects and improves endurance in the HZSO|HZO films. b) $E_{c}$ and fatigue cycles reported in literature for similar thickness of epitaxial HfO_2_‐based thin films with different polar phases. Blue region corresponds to *r*‐d *o*‐phase, brown region corresponds to *r*‐phase, and purple region corresponds to *o*‐phase. The red square corresponds to HZO and the green star corresponds to HZSO|HZO from this work. The fatigue from multiple doped‐HfO_2_ works is extracted from when $P_{r}$ is less than 80\% of initial $P_{r}$.^[^ ^14^, ^16^, ^18^, ^23^, ^37^, ^38^, ^39^, ^40^, ^41^, ^42^, ^43^ ^]^

In **Figure** **4**a, schematics of HZO and HZSO|HZO with bottom LSMO and top Pt electrodes are shown. In the HZO film, oxygen redistribution in HZO is triggered by an external electric field, facilitating oxygen exchange between LSMO and HZO. As noted earlier, the insertion and extraction of oxygen into the HZO is crucial for achieving high endurance. In the HZO, V_O_ are linked to cation redox, in contrast to the structural V_O_ in HZSO. With the HZSO seed layer, as demonstrated by HAXPES (Figure 3e) and shown schematically in Figure 4a, there is easier reversible extraction and insertion of oxygen ions between HZSO and LSMO. The fact that HZSO and HZSO|HZO show the same fatigue behavior indicates the critical role of interfacial O exchange with LSMO on fatigue behavior (see Figure S4, Supporting Information). At the same time, a lower E_c_ in HZSO is achieved through a proximity effect with HZO layer. Our strategy of using a novel ionic conductor underlayer of HZSO enables a lower E_c_ and significantly improved endurance compared to HZO without compromising its P_r_. Furthermore, the chemical and structural compatibility of HZSO and HZO ensures a high quality and stable interface which is critical for reproducible device behaviour and long‐term stability.

Figure 4b summarizes the performance of the devices of this work to literature. E_c_ and fatigue cycles of previously reported epitaxial HfO_2_‐based thin films with *r*‐d *o*‐, *o*‐, and *r*‐phases are shown. It is reiterated that for a more stringent comparison, fatigue is defined as the number of cycles required for P_r_ to be less than 80% of the initial P_r_. Epitaxial films typically demonstrate high E_c_, especially *r*‐d *o*‐ and *r*‐phases. We observe that our HZSO|HZO film shows enhanced endurance while also having a relatively low E_c_ value amongst epitaxial HfO_2_‐based thin films, such a combination not having been achieved in the other reported films. Thus, in the presence of a redox‐free ionic conductingintrinsically polar HZSO underlayer, HZO films are engineered with a simultaneous improvement of both E_c_ and endurance performance, which is important for future low‐power memory devices.

## 3 Conclusion

In conclusion, in HZO thin films, we report a novel approach to reduce E_c_ and improve endurance that is applicable to an industry‐compatible process (e.g. ALD). Our strategy is to use an ultrathin (≈2 nm) 5 at.% Sm‐doped HZO (HZSO) ionic conducting, non‐redox exhibiting source/sink underlayer in which structural V_O_ are present. The HZO is epitaxially grown in the rhombohedrally‐distorted orthorhombic (*r*‐d *o*‐) phase on top of the underlayer. From Nudge Elastic Band calculations, it is shown that E_c_ in HZSO can bes decreased in the presence of Sm and structural V_O_. The concomitant reduction of E_c_ in the HZSO|HZO films can be explained by a proximity effect with the HZSO layer. An order of magnitude improvement of fatigue in the HZSO|HZO films is achieved, consistent with the control of the insertion and extraction of oxygen ions in the HZSO film by the LSMO due to the ionic conductor characteristics of the HZSO layer. Overall, by introducing a novel underlayer approach in HZO thin films, this work opens new avenues for improving the key ferroelectric properties in HfO_2_‐based thin films for memory applications.

## 4 Experimental Section

### Deposition of Thin Films

To make the HZO target, HfO_2_ and ZrO_2_ powders were ground and mixed for an hour and compressed into a pellet, which was sintered for 8 h at 1400 °C. HZSO target was fabricated with appropriate ratios of HfO_2_, ZrO_2_, and Sm_2_O_3_ powders, using the same sintering process as HZO. LSMO target was fabricated with LaCO_3_, SrCO_3_, and MnO powders, which were first calcined at 850 °C and then sintered at 1200 °C. LSMO buffer layers were grown at 750 °C under 100 mTorr (0.133 mbar) oxygen partial pressure with a laser fluence of 0.7 J cm^−2^ and a laser frequency of 2 Hz. The number of unit cells for LSMO was tracked using reflection high energy electron diffraction (RHEED). Epitaxial 10‐nm‐thick films of Hf_0.53_Zr_0.47_O_1.72_ (HZO) and Hf_0.5_Zr_0.45_Sm_0.05_O_1.61_ (HZSO) were grown with 1.3 J cm^−2^. HZSO seed layer was grown with 0.5 J cm^−2^. They were grown at 890 °C under 75 mTorr (≈0.1 mbar) oxygen atmosphere with a laser frequency of 1 Hz. All the samples were grown using PLD with a KrF excimer laser (λ = 248 nm). The spot size was kept constant at 2.5 mm^−2^ for all the depositions with target to substrate distance of 5 cm. After deposition, the heterostructures were cooled to room temperature at a rate of 5 °C min^−1^ under 0.4 bar of oxygen partial pressure.

### Characterization of Thin Films

A PANalytical Empyrean Diffractometer was used for the XRD characterization. STEM was conducted using a Thermo Fisher Scientific Spectra 300. For HAXPES, Ga Kα (9.25 keV) metal jet source in the Henry Royce Facility of the Photon Science Institute at the University of Manchester. A ≈50 μm focused beam was aligned to the poled regions of interest at a 30°. Photoelectrons were collected in an EW4000 energy analyzer (ScientaOmicron GmbH) at an electron take‐off angle of 60° to gain sensitivity to shallower film depths while maintaining a <300‐μm‐diameter beam spot. A pass energy of 500 eV and a slit size of 0.8 mm was utilized to optimize for signal and energy resolution. Hf‐3d, O‐1s, and Zr‐2p core‐levels were probed. The samples were grounded to the LSMO bottom contact and no charging was observed during the measurement.

For in situ XPS, samples were transferred in situ from the PLD chamber to an attached XPS analysis chamber. An Al Kα X‐ray source and a SPECS PHOIBOS 150 hemispherical analyzer were used to collect high resolution Sm‐3d and C‐1s core‐level spectra.

For electrical measurements, 50 μm‐diameter electrodes (tungsten and platinum) were patterned using UV lithography mask. Top electrodes were sputtered using DC sputtering. Characterization of ferroelectricity was conducted using AixACCT TF analyzer 2000. Piezo‐response force microscopy (PFM) was performed using a Bruker Multimode 8 atomic force microscope with Pt‐coated NSC35 tips (MikroMasch) with spring constant of 5.4 N m^−1^. The sample was mounted on metallic disc where V_dc_ was applied via bottom LSMO while grounding the top Pt tip. PFM poling was detected using V_ac_ of 1 V at 10 kHz.

### Nudge Elastic Band Calculations

A 2x2x2 supercell of rhombohedrally distorted o‐phase HZO (α = β = γ = 89.07°), containing 96 atoms, was built with alternating Hf and Zr planes along the (001) direction.^[^ ^67^ ^]^ Two Hf atoms were substituted with Sm, corresponding to a doping concentration of 6.125 cat.%. The defects were ionically compensated by introducing a single oxygen vacancy. This choice ensured charge compensation of the system.^[^ ^68^ ^]^ The V_O_ and dopants were chosen among low‐energy configurations.^[^ ^69^ ^]^ For comparison purposes, an electronically compensated cell and an undoped cell with a single neutral oxygen vacancy were also built.

All calculations were performed with VASP 6.4.1^[^ ^70^, ^71^, ^72^, ^73^ ^]^ at the GGA level of theory, using the PBESol functional.^[^ ^74^ ^]^ A 2x2x2 K‐point grid and a 600 eV plane wave energy cutoff was used for relaxing the structures. Switching energy barriers were calculated using the climbing image nudged elastic band (CI‐NEB) method^[^ ^75^ ^]^ with a force convergence threshold of 10 meV Å^−1^ and using a minimum of 11 images per calculation. The atomistic representations were generated using VESTA.^[^ ^76^ ^]^

## Conflict of Interest

The authors declare no conflict of interest.

## Author Contributions

J.S.K. and J.L.D. conceived the idea and the project plan. J.S.K. fabricated samples and performed structural and ferroelectric properties characterization with the help from N.S. B.B. performed RBS. B.G. and J.S.K. performed in‐situ XPS and J.S.K. analyzed the data. S.M.F. performed cross‐sectional STEM. V.L. and L.M. carried out NEB calculations. J.L.D. and J.P.B.S. supervised the project. All authors discussed the experimental data. J.S.K., J.L.D., and J.P.B.S. co‐wrote the manuscript with feedback from all the authors.

## Supporting information

Supporting Information

## Data Availability

The data that support the findings of this study will be deposited in the University of Cambridge Data Repository (Apollo) and will be made openly available with a DOI upon acceptance of this manuscript.
