# Supplementary material for: Reducing Coercive Field and Improving Endurance in Ferroelectric Epitaxial Hf0.5Zr0.5O2 Thin Films via Novel Interface Layer Approach
Source: Adv Sci (Weinh). 2025 Nov 14;13(5):e17314. doi: 10.1002/advs.202517314 (PMC12850052; doi:10.1002/advs.202517314)
Supplement: Supplementary file 1 — Supporting Information [file ADVS-13-e17314-s001.pdf]

Supplementary Information: Reducing coercive field and improving endurance in ferroelectric epitaxial $Hf_{0.5}Zr_{0.5}O_2$ thin films via novel interface layer approach

Ji Soo Kim[1,⋆], Benedetta Gaggio[1], Babak Bakhit[1], Veniero Lenzi[2,3], Luis Marques[2,3], Simon M. Fairclough[1], Nives Strkalj[1,†], Duk-Hyun Choe[4], José P. B. Silva[2,3,⋆], and J. L. MacManus-Driscoll[1,⋆]

[1] *Department of Materials Science & Metallurgy, University of Cambridge, 27 Charles Babbage Road, Cambridge CB3 0FS, United Kingdom*

[2] *Physics Center of Minho and Porto Universities (CF-UM-UP), University of Minho, Campus de Gualtar, 4710-057 Braga, Portugal*

[3] *Laboratory of Physics for Materials and Emergent Technologies, LapMET, University of Minho, 4710-057 Braga, Portugal*

[4] *Samsung Advanced Institute of Technology, Samsung Electronics, Suwon-si, 16678, Korea*

[⋆] *Correspondence should be sent to: Ji Soo Kim (jsk55@cam.ac.uk) or José P.B. Silva (josesilva@fisica.uminho.pt) or Judith L. MacManus-Driscoll (jld35@cam.ac.uk)*

[†] *Present address: Center for Advanced Laser Techniques, Institute of Physics, 10000 Zagreb, Croatia*

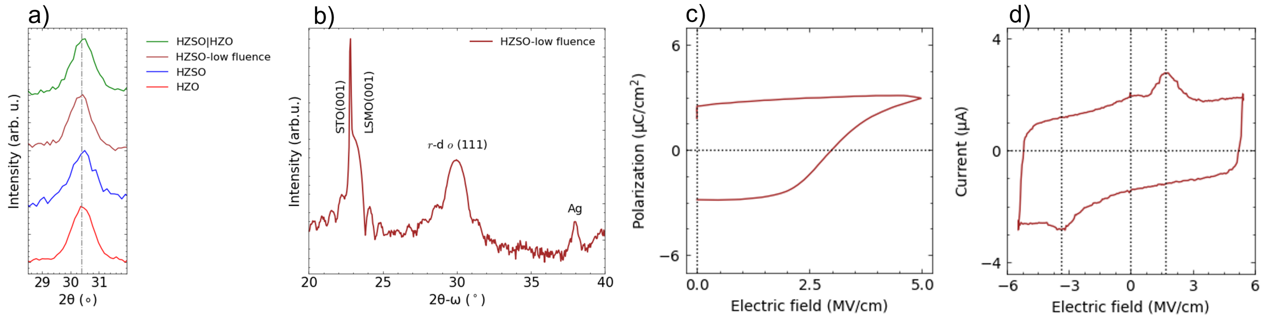

Figure S 1: a) 2θ measurements at χ∼70° and ϕ∼45° on HZO, HZSO (varying laser fluences) and HZSO|HZO thin films. Peaks were found at 2θ∼30.5°, indicative of rhombohedral distortion. b) 2θ-ω measurement on HZSO grown at lower laser fluence (0.5 J/cm^2). A clear r-d o(111) peak was identified. Additional peaks originate from silver paste. c) P-E loop and d) I-E loops of the HZSO. Its P_r and E_c are ∼3 μC/cm^2 and ∼2.6 MV/cm, respectively and are identical to HZSO grown at higher laser fluence (1.3 J/cm^2), indicating that laser fluence does not influence ferroelectric properties of HZSO.

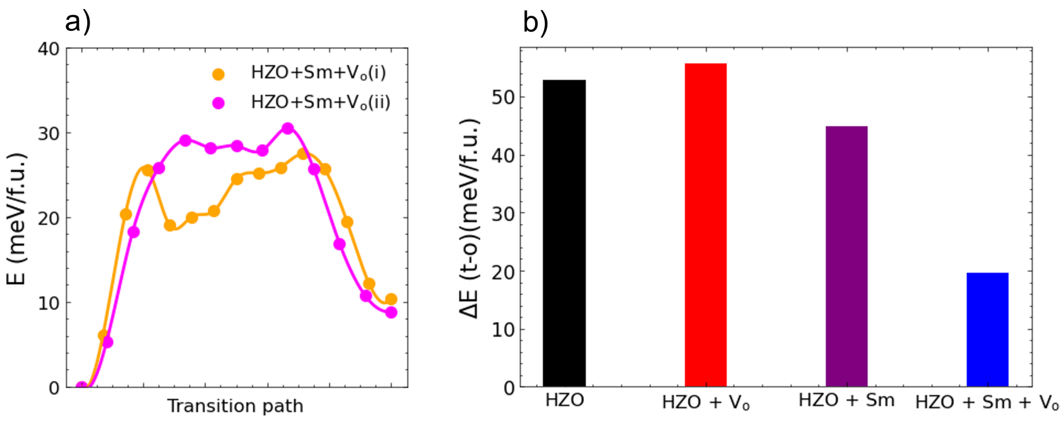

Figure S 2: a) Nudge elastic band (NEB) calculations on HZO+Sm+V_O for two different locations of V_O, denoted as (i) and (ii) showing similar intermediate energy barrier. b) Calculated relative energy, ΔE (t-o), between the intermediate t-phase (e.g. $P4_2/nmc$) and the rhombohedrally distorted o-phase, obtained under different conditions. HZO+Sm+$V_O$shows a reduced energy barrier compared to HZO and HZO+V_O.

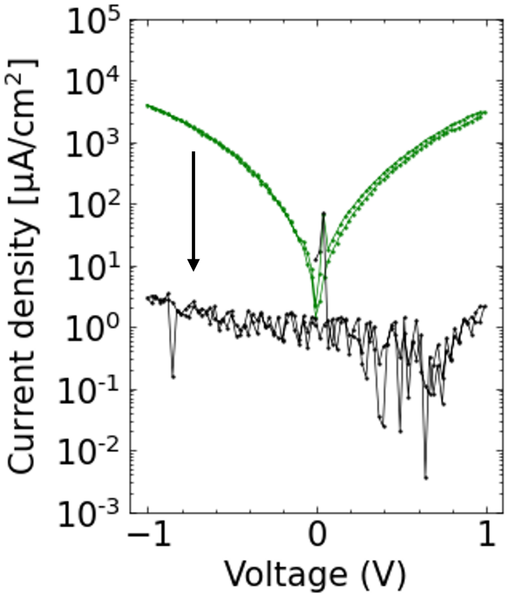

Figure S 3: Leakage current measurement on HZSO|HZO pre- (green) and post-fatigue (black) at 5×10^8 cycles using ±1V. A significant reduction in leakage current is observed.

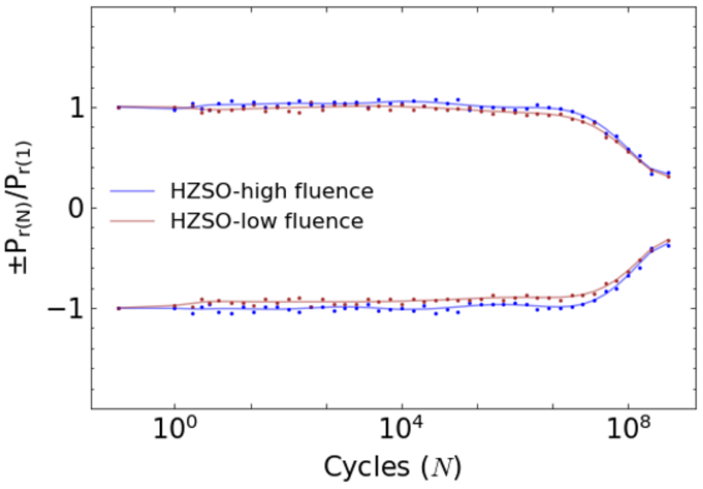

Figure S 4: Endurance measurements on HZSO grown at different laser fluence showing ferroelectricity degradation.

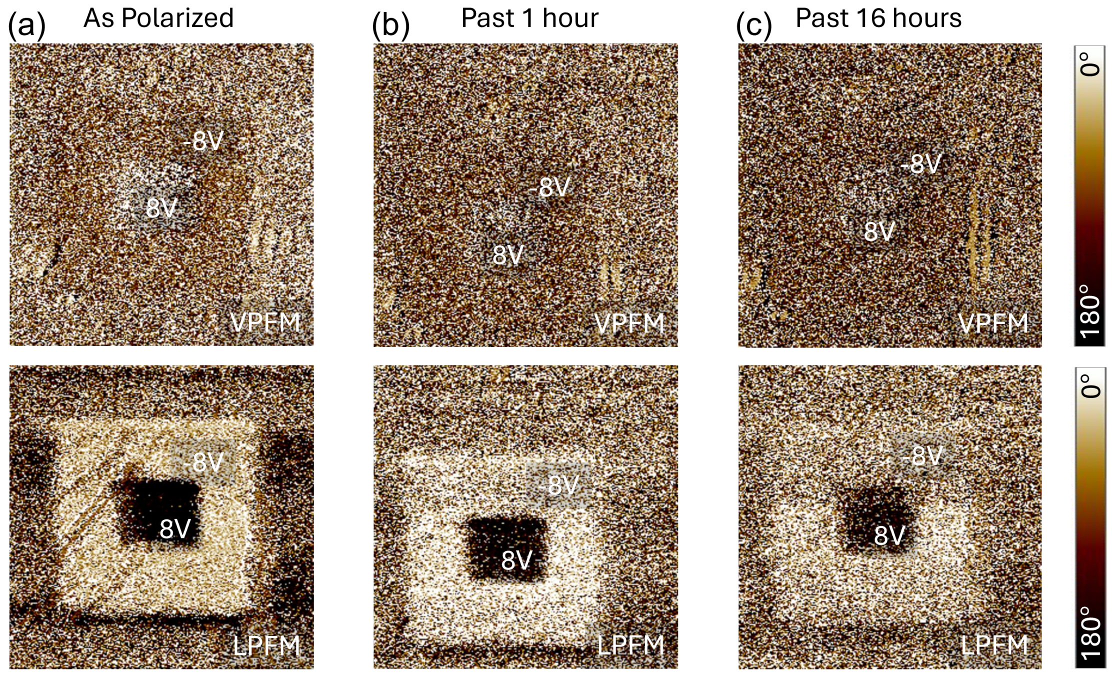

Figure S 5: PFM: vertical and lateral phase response measurements of HZSO sample at a) as polarized, b) 1 hour after polarization and c) 16 hours after polarization show HZSO retains its polarization for more than 16 hours.

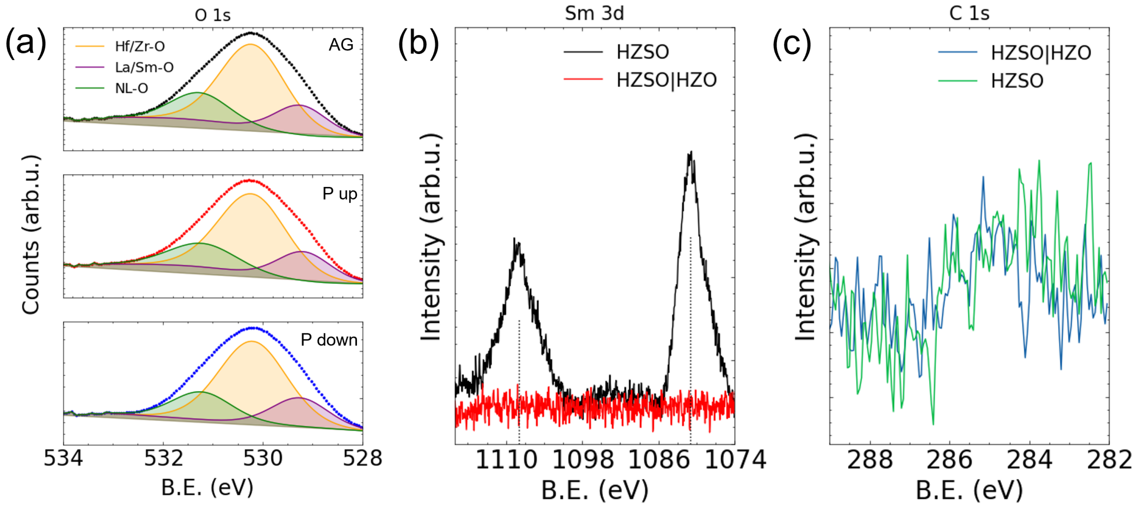

Figure S 6: O-1s core-level spectra from HAXPES on HZSO sample. Any change between AG, P up and P down are within the resolution limit. b) In-situ XPS probing Sm-3d core-level spectra for HZSO and HZSO|HZO. Clear doublet of Sm-3d peaks were found from HZSO, but not from HZSO|HZO, indicating Sm confinement in the seed layer. c) C-1s core-level was probed to show absence of a peak, indicative of absence of adsorbed organic species during in-situ transfer.

	NL-O	Hf/Zr-O	La/Sm-O	Hf 3d FWHM	Zr 2p FWHM
AG	18.62%	63.19%	18.19%	2.91506	2.68538
P up	18.58%	63.08%	18.34%	2.84399	2.75854
P down	18.39%	63.22%	18.39%	2.90034	2.69951

Table S 1: Fitted results of O-1s core-level spectra from HAXPES measurement. No significant change was observed in the ratio of Hf/Zr-O, La/Sm-O and NL-O
